# The interplay of oxidative stress and ARMS2-HTRA1 genetic risk in neovascular AMD

**DOI:** 10.20517/2574-1209.2020.48

**Published:** 2021-01-15

**Authors:** Zhi-Gang Lu, Adam May, Brian Dinh, Victor Lin, Fei Su, Christina Tran, Harini Adivikolanu, Rachael Ehlen, Briana Che, Zhi-Hao Wang, Daniel H. Shaw, Shyamanga Borooah, Peter X. Shaw

**Affiliations:** 1 Department of Neurology, First People’s Hospital of Jingmen, Jingchu University of Technology, Jingmen 448000, Hubei, China; 2 Viterbi Family Department of Ophthalmology and Shiley Eye Institute, University of California, San Diego, La Jolla, CA 92093, USA; 3 Altman Clinical and Translational Research Institute, University of California, San Diego, La Jolla, CA 92093, USA; 4 Westview High School, San Diego, CA 92131, USA

**Keywords:** Neovascular AMD, oxidative stress, inflammation, polymorphism of HTRA1 gene

## Abstract

Age-related macular degeneration (AMD) is the leading cause of vision loss in adults over 60 years old globally. There are two forms of advanced AMD: “dry” and “wet”. Dry AMD is characterized by geographic atrophy of the retinal pigment epithelium and overlying photoreceptors in the macular region; whereas wet AMD is characterized by vascular penetrance from the choroid into the retina, known as choroidal neovascularization (CNV). Both phenotypes eventually lead to loss of central vision. The pathogenesis of AMD involves the interplay of genetic polymorphisms and environmental risk factors, many of which elevate retinal oxidative stress. Excess reactive oxygen species react with cellular macromolecules, forming oxidation-modified byproducts that elicit chronic inflammation and promote CNV. Additionally, genome-wide association studies have identified several genetic variants in the age-related maculopathy susceptibility 2/high-temperature requirement A serine peptidase 1 (ARMS2-HTRA1) locus associated with the progression of late-stage AMD, especially the wet subtype. In this review, we will focus on the interplay of oxidative stress and HTRA1 in drusen deposition, chronic inflammation, and chronic angiogenesis. We aim to present a multifactorial model of wet AMD progression, supporting HTRA1 as a novel therapeutic target upstream of vascular endothelial growth factor (VEGF), the conventional target in AMD therapeutics. By inhibiting HTRA1’s proteolytic activity, we can reduce pro-angiogenic signaling and prevent proteolytic breakdown of the blood-retina barrier. The anti-HTRA1 approach offers a promising alternative treatment option to wet AMD, complementary to anti-VEGF therapy.

## INTRODUCTION

Age-related macular degeneration (AMD) is the leading cause of irreversible central vision loss in people over 60 years old^[1]^ and is a burgeoning public health problem in many developed countries due to aging populations^[2]^. There are two types of AMD: dry AMD, characterized by geographic cellular atrophy in the macula; and wet AMD, a proliferative retinopathy caused by aberrant blood vessel growth from the choroid into the neural retina^[3]^. While wet AMD only accounts for 10%–15% of patients with AMD, it is responsible for 90% of AMD-related severe visual impairment^[4]^. Despite recent advances in understanding the mechanisms of AMD progression, it remains unclear what predisposes individuals to develop dry *vs*. wet AMD and what triggers the transition from dry to wet AMD in certain patients. Finding answers to these questions will not only catalyze the development of more effective therapeutics against AMD but may also elucidate mechanisms underlying other neovascular diseases.

Like many age-related diseases, AMD is believed to result from an interplay of genetic, environmental, and behavioral risk factors. The complex etiology of AMD closely resembles that of polypoidal choroidal vasculopathy (PCV) compared to other retinopathies [Table 1]. However, the choroidal neovascularization (CNV) associated with wet AMD does not display the hallmark vascular polyp-like dilations found in patients with PCV, distinguishing the two conditions. The primary environmental/behavioral risk factor for AMD is cigarette smoking, likely *via* an increase in systemic oxidative stress that promotes chronic inflammatory responses in the retina^[23–25]^. Chronic retinal inflammation is thought to accelerate degradation of the blood-retina barrier and trigger signaling cascades that increase pro-angiogenic gene expression associated with wet AMD^[26]^. The most prominent genetic risk factors for AMD include the nonsynonymous variant rs1061170 on chromosome 1q31, which encodes a Tyr402His substitution in complement factor H (CFH), and an ~5-kb high linkage-disequilibrium (LD) block spanning the age-related maculopathy susceptibility 2/high-temperature requirement A serine peptidase 1 (ARMS2-HTRA1) locus on chromosome 10q26^[27–30]^. Because the ARMS2-HTRA1 risk haplotype slightly favors progression to wet AMD, we focus this review on the interplay among ARMS2-HTRA1 genetic risk, cigarette smoking, and oxidation-induced inflammatory responses on wet AMD development. We conclude with a mechanistic model of wet AMD progression that we hope will stimulate future investigations on wet AMD and other neovascular diseases.

### PATHOLOGY OF WET AMD

**Figure 1.**
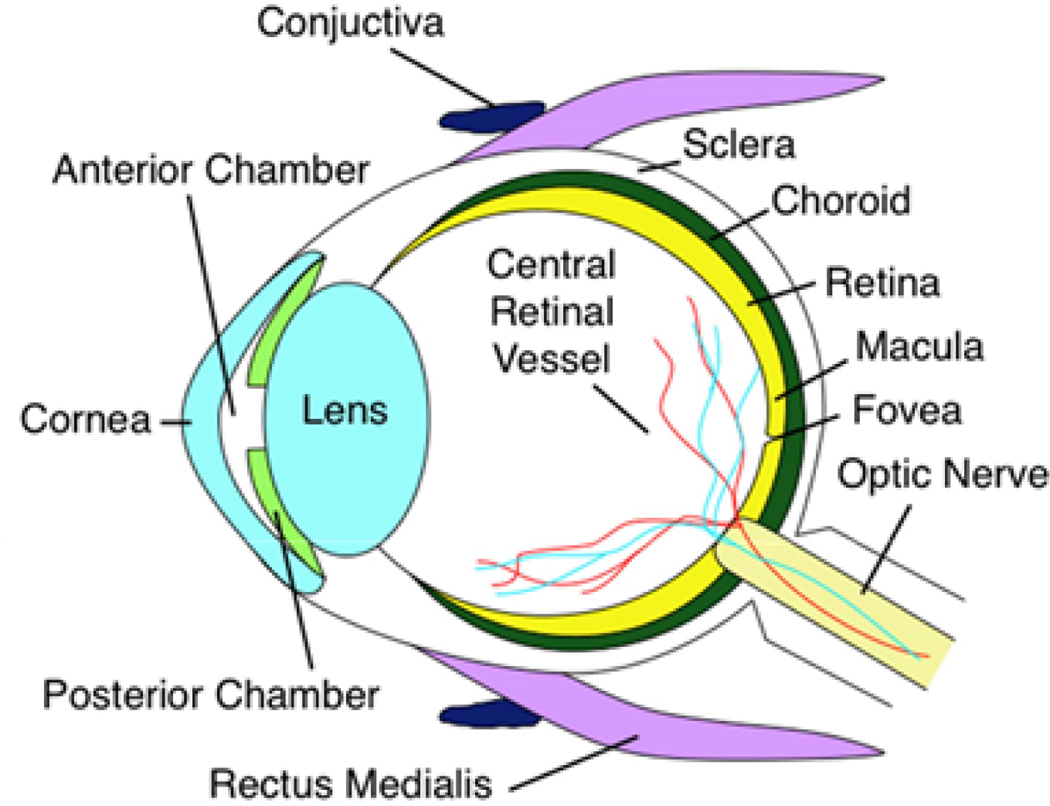
Anatomical cross section of the human eye. The macula is the central portion of the retina, responsible for high-acuity vision. Age-related macular degeneration progression leads to macular dystrophy, death of overlying photoreceptors, and eventually central vision loss

**Figure 2.**
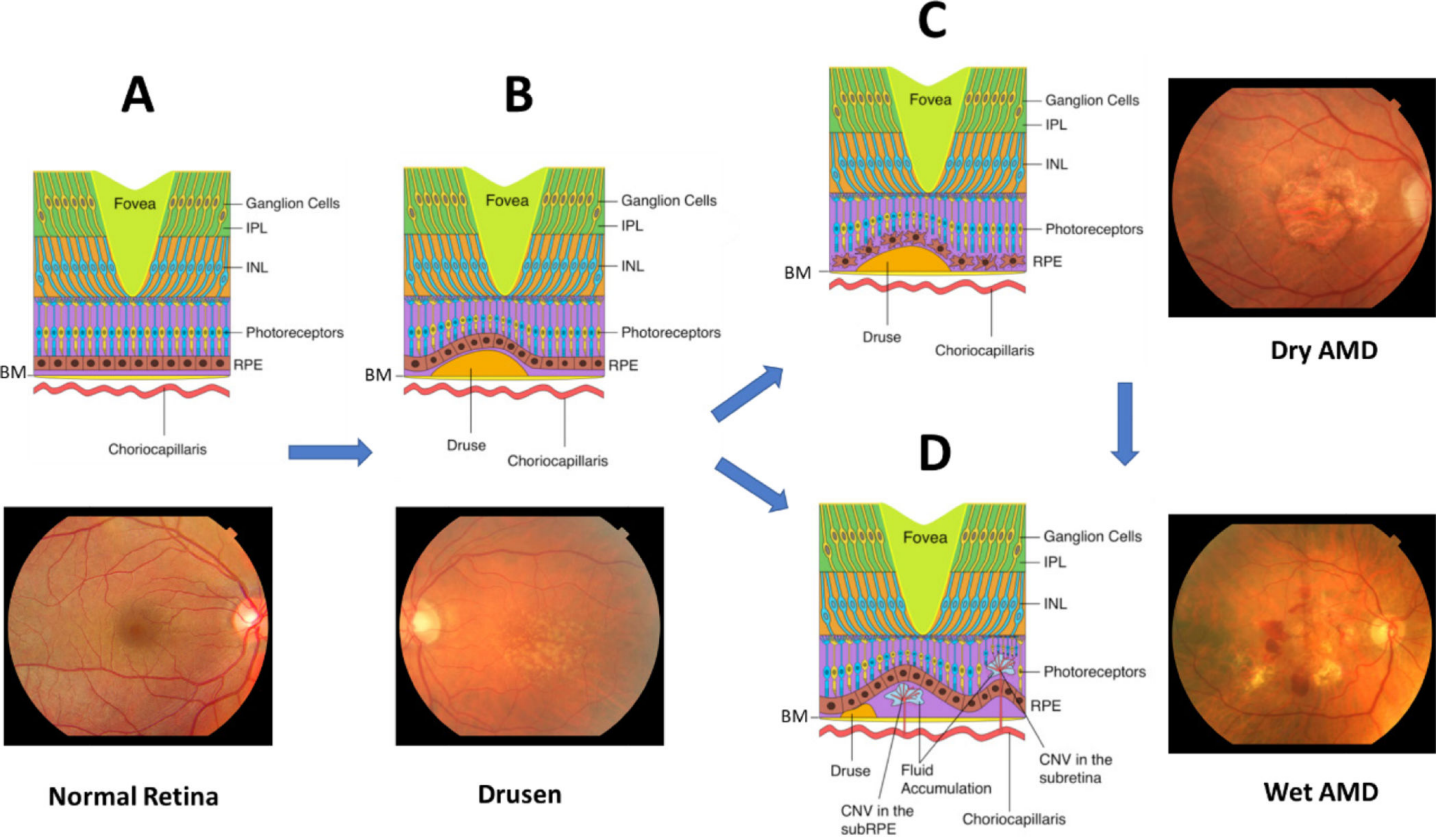
The clinical features of AMD progression. (A) Cross section and fundus photo of normal retina showing BM; choriocapillaris; IPL; INL; photoreceptors; and RPE; (B) drusen form underneath the RPE cell layer, preceding late-stage AMD; (C) dry AMD is characterized by extensive drusen, atrophy of RPE and photoreceptors, and deformed blood-retina barrier; (D) wet AMD is characterized by choroidal neovascularization across Bruch’s Membrane into the macula, leading to subretinal hemorrhage and possibly retinal detachment. AMD: age-related macular degeneration; BM: Bruch’s Membrane; IPL: inner plexiform layer; INL: inner nuclear layer; RPE: retinal pigment epithelium

AMD leads to degeneration of the macula, the cone-rich central portion of the retina that is adapted for high-acuity daytime color vision [Figure 1]^[31]^. It primarily affects the blood-retina barrier, consisting of the retinal pigment epithelium (RPE) and underlying Bruch’s Membrane (BM), and secondarily the photoreceptors [Figure 2A]. Proper phototransduction by photoreceptors is reliant on an intact and functional blood-retina barrier for uptake of essential nutrients from the choroidal vasculature, disposal of cellular waste to systemic circulation, and regeneration of the active chromophore 11-*cis*-retinal *via* the visual cycle, among other functions^[32,33]^. This delicate balance is disrupted in the AMD eye. The first clinical manifestations of disease are drusen, which appear on color fundus as yellowish focal deposits beneath the retina [Figure 2B and C]. They accumulate in the extracellular space between the RPE and BM and have recently been suggested to result from the oligomerization of lipids and proteins onto hydroxyapatite spherules^[34]^. Regardless of their origin, the size and number of drusen are used to determine an individual’s disease stage and assess the likelihood of progression to advanced AMD with severe central vision loss^[35,36]^. “Soft” drusen with poorly demarcated boundaries or diameter greater than 125 μm are particularly prognostic for severe AMD^[37,38]^. Unfortunately, drusen characteristics do not readily distinguish between individuals who will develop dry *vs.* wet AMD^[38]^.

In the early stages of wet AMD, inflammatory cells are observed in the subretinal space between the RPE and BM^[39]^. They disrupt the integrity of the blood-retina barrier through the release of inflammatory oxidants and pro-angiogenic cytokines and chemokines, including vascular endothelial growth factor (VEGF). VEGF stimulates endothelial cells to proliferate, migrate, and germinate to form new capillaries that cross the BM into the neural retina, a process referred to as CNV [Figure 2D]^[40,41]^. VEGF mediates the expression of MCP-1, an angiogenic chemokine that recruits more macrophages to the retina^[42,43]^, and acts as a specific endothelial cell mitogen to increase vascular permeability^[44]^. The structural integrity of the blood-retina barrier is further compromised by macrophage matrix metalloproteinases (MMP), which degrade the pentalaminar fibrous proteins of the BM^[45–49]^. As CNV expands within the subretinal space, the macula deforms and subretinal hemorrhaging allow cells and fluid in circulation to leak into the neural retina^[50]^. In its most advanced stages, CNV sites become fibrotic and form disciform scars, leading to necrosis of RPE and photoreceptors^[33,50]^. Photoreceptor cell death secondary to these processes is the immediate cause of vision loss for the patient.

### REDOX BIOCHEMISTRY AND OXIDATIVE STRESS IN THE RETINA

#### Introduction to oxidation in the retina

Free reactive oxygen species (ROS) can react with biological macromolecules to form oxidation-modified, pro-inflammatory species^[51,52]^. Photoreceptor outer segments are rich in polyunsaturated fatty acids, which are easily oxidized by photosensitizers in the retina^[53]^. As these highly oxidized outer segments are shed, they are phagocytosed by the RPE through a tightly regulated sequence of steps that involves outer segment recognition and binding by the integrin αvβ5^[54,55]^ and the scavenger receptor CD36^[56]^, followed by activation of MERTK to trigger internalization^[57]^. Once inside the RPE, the outer segments are degraded along the phagolysosomal pathway into compounds that are either recycled back into the visual cycle or exocytosed from the RPE basolateral membrane into the choroid for clearance from systemic circulation^[32]^. The continuous shedding of oxidized outer segments is a major phagocytic challenge for the RPE that heightens intracellular oxidative stress and may contribute to age-related accumulation of oxidation-modified lipids and proteins in the retina, leading to disease^[58,59]^.

#### The inflammatory response to oxidative stress

As people age, the permeability of Bruch’s Membrane decreases, thereby hindering the transport of exocytosed waste products from the RPE to the choroid^[60]^. These waste products accumulate in the sub-RPE space, prolonging their exposure to the oxidizing extracellular environment. Oxidation-specific epitopes, also referred to as Damage-Associated Molecular Patterns (DAMPs), on otherwise normal lipids and proteins, signal to the cell that the parent compound is damaged, flagging it for clearance by the immune system^[61]^. Many cytokines and chemokines are recruited during the inflammatory response, promoting angiogenesis and CNV [Table 2].

DAMPs are bound by a variety of Pattern Recognition Receptors (PRRs), including scavenger receptors, toll-like receptors, C-reactive protein, complement components, and IgM antibodies^[80,81]^. Interestingly, drusen have also been found to contain C-reactive protein, immunoglobulins, and many components of the complement pathway^[82,83]^. The presence of these complement proteins and oxidation products has been shown to result in changes to the cellular composition of the RPE and choroid. However, before these changes occur, cellular oxidative stress must elicit the secretion of pro-inflammatory cytokines and chemokines, overwhelming the cellular regulatory mechanisms. In the next section, we will review how this redox regulatory system becomes overburdened in the AMD eye.

#### Overview of redox biochemistry

Biochemical oxidation-reduction reactions are central components of many metabolic and signaling pathways^[84]^. As such, they are tightly regulated using a variety of enzymatic and non-enzymatic mechanisms, which control the balance of pro-oxidant and antioxidant species^[52]^. One of the most studied redox regulators is glutathione (GSH), a tripeptide of glutamate, cysteine, and glycine^[85]^. When exposed to oxidizing agents, glutathione’s thiol moiety is oxidized to form a disulfide bridge with another molecule of glutathione, forming glutathione disulfide (GSSG)^[86]^. GSSG can be reduced by glutathione reductase (GSH reductase) at the expenditure of one NADPH, reforming two GSH^[86]^. Together, the interconversion of GSH and GSSG forms a redox buffer system^[87]^, analogous to the bicarbonate buffer system for pH.

**Figure 3.**
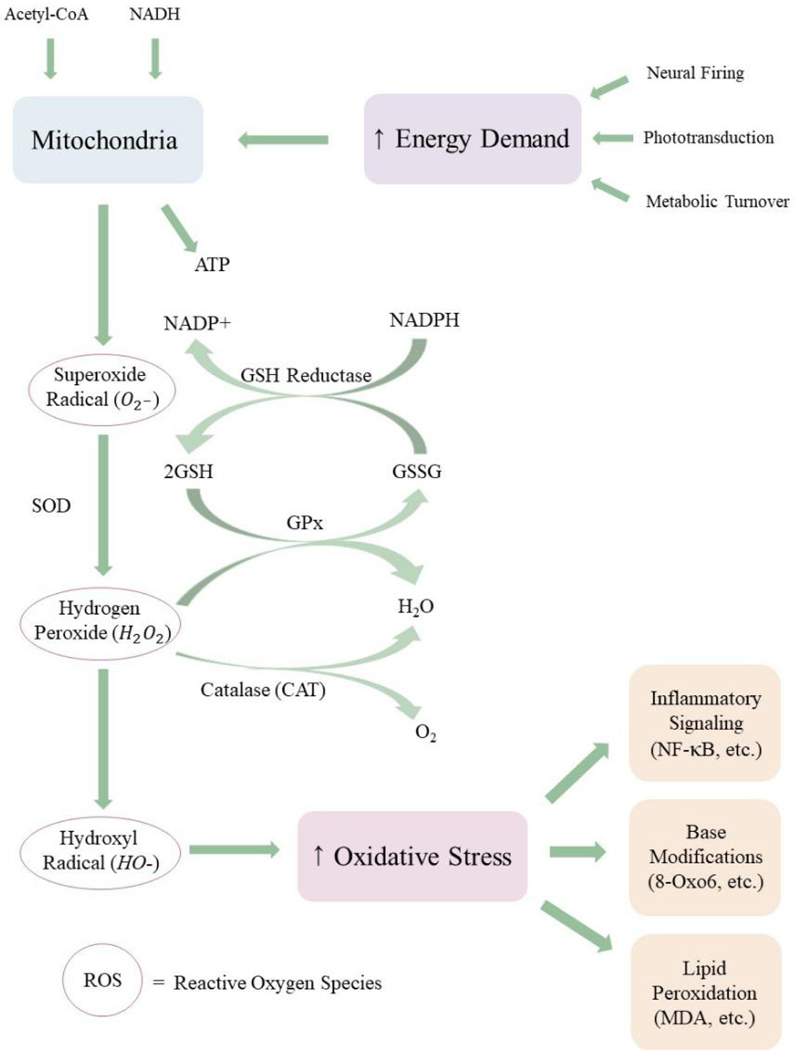
The flow of ROS in the retina. Mitochondria respond to high energy demand in the retina by synthesizing ATP, a by-product of which is the potent ROS superoxide radical. Superoxide radical is converted to less reactive species through a sequence of enzymatic and non-enzymatic pathways. If mitochondrial ROS release exceeds antioxidant scavenging capacity, ROS accumulate in the retina and can react with macromolecules, including DNA and unsaturated phospholipids. Elevated ROS and oxidation-modified by-products elicit chronic inflammation, contributing to retinal pathologies like AMD. ROS: reactive oxygen species; AMD: age-related macular degeneration

The status of the redox buffer system can be used to assess the oxidative state of the intracellular environment. Studies have shown that the GSH:GSSG ratio remains within narrow ranges that are specific to particular intracellular compartments^[88]^. The GSH:GSSG ratio of the endoplasmic reticulum ranges from 1:1 to 1:3^[89,90]^, whereas the GSH:GSSG ratio of the cytosol ranges from 30:1 to 100:1^[90]^. However, chronic overproduction of oxidizing agents can alter redox homeostasis and decrease the GSH:GSSG ratio, which has been correlated with aging and disease^[91,92]^. The greatest endogenous source of oxidizing agents is release of ROS from the mitochondrial matrix as byproducts of oxidative phosphorylation^[93]^. As electrons move along the electron transport chain, some electrons leak from Complexes I and III, forming superoxide radical (O_2_^-^)^[94]^. Superoxide radical is scavenged by superoxide dismutase (SOD) to form hydrogen peroxide (H_2_O_2_), which may be either reduced to water by glutathione peroxidase (GPx) at the expenditure of one glutathione or dismutated to water and molecular oxygen by catalase (CAT)^[95]^. However, excess leakage of O_2_^-^ can overload these enzymatic mechanisms, resulting in increased ROS concentration in the cytosol and nucleus [Figure 3]. Excess ROS can lead to nuclear and mitochondrial DNA damage, autophagy decay, and apoptosis of photoreceptors and RPE *via* the MAPK/ERK 1/2 pathway^[96–99]^. In addition to an endogenous leakage of mitochondrial ROS, cigarette smoke, a prominent environmental/behavioral risk factor for AMD^[100–103]^, contains an assortment of free radicals and ROS that disturb cellular redox^[104]^.

#### Oxidative stress in the retina

The retina is especially prone to increased endogenous ROS production due to its high energy demand, high dissolved oxygen concentration, and dependency on mitochondrial oxidative phosphorylation for proper function^[105,106]^. Photoreceptor mitochondria are predominantly clustered within the inner segment, whereas the outer segment is sparsely populated^[107]^. Unlike most neurons, photoreceptors are depolarized in their unstimulated state and are hyperpolarized upon stimulation with light, forming the dark current^[108]^. In the absence of light, Na^+^/K^+^ ATPases are actively maintaining a continual flux of Na^+^ into the cell, enabling continual release of the excitatory neurotransmitter glutamate into the photoreceptor-bipolar cell synaptic cleft^[109]^. This ATPase activity accounts for most retinal energy requirements^[109,110]^. The RPE is also densely populated with mitochondria, which are used to power phagocytosis of photoreceptor outer segments; metabolic exchange between the neural retina and the choroid; and maintenance of transepithelial potential^[107,111–113]^.

### ARMS2-HTRA1 GENETIC RISK AND HTRA1 ACTIVITY IN WET AMD Introduction to ARMS2-HTRA1 in AMD: discovery and controversy

**Figure 4.**
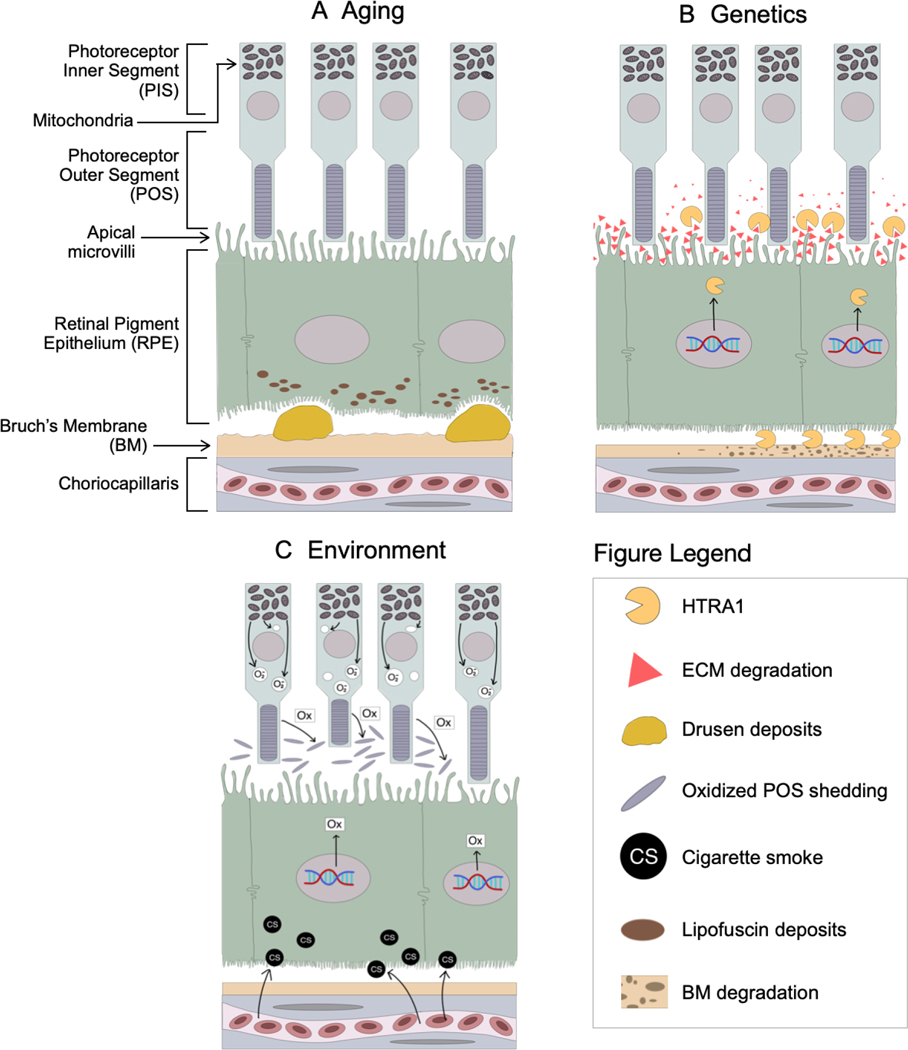
Retinal impact of wet AMD risk factors. (A) Depicts age-related lipofuscin accumulation and drusen formation; (B) the ARMS2-HTRA1 locus is a prominent genetic risk factor for AMD, resulting in excess HTRA1 expression and heightened degradation of RPE extracellular matrix and Bruch’s Membrane; (C) oxidative stress due to mitochondrial superoxide release and bloodborne cigarette smoke extracts alter transcriptional pathways that increase oxidative load in the retina. AMD: age-related macular degeneration; RPE: retinal pigment epithelium

In addition to cigarette smoking and oxidative stress, many genetic variants have been linked to AMD pathogenesis. The first genome-wide association studies (GWAS) on AMD identified variants in the complement factor H (CFH) locus on chromosome 1q31 that strongly associate with progression to late-stage AMD^[114–117]^. Shortly thereafter, two studies simultaneously discovered another strong genetic risk locus on chromosome 10q26^[118,119]^. These risk variants span a ~5-kb region of high linkage disequilibrium (LD) that includes the promoter of HtrA serine peptidase 1 (HTRA1), a secreted serine protease that has been extensively studied for its role in extracellular matrix (ECM) remodeling^[120–122]^, transforming growth factor β (TGF-β) signaling^[123–127]^, and various cancers^[128–130]^. Preliminary evidence from luciferase reporter assays using HTRA1 promoter sequences^[118]^ and RT-PCR of HTRA1 mRNA from blood lymphocytes^[119]^ indicated that the AMD risk haplotype increases HTRA1 expression ~2-fold. These findings support a putative disease mechanism in which chronically elevated HTRA1 due to the risk haplotype alters ECM homeostasis along the blood-retina barrier, leading to AMD. However, the risk haplotype also extends into the coding sequence for age-related maculopathy susceptibility 2 (ARMS2), an 11-kDa protein whose structure, function, and localization have been disputed since its discovery. Initially thought to be a mitochondrial protein^[131,132]^, ARMS2 is now known to be secreted from cells^[133]^ *via* a Golgi-independent pathway^[134]^ and may regulate complement activation^[135]^. However, ARMS2 seems to be an unlikely source for AMD pathogenicity due to (1) its weak expression in the retina^[131,132,136,137]^ and (2) a recent expression quantitative trait loci (eQTL) analysis that found multiple AMD-associated eQTLs that affect HTRA1 expression, but *no* AMD-associated eQTLs that exclusively affect ARMS2 expression^[138]^. While it is our view that HTRA1 is the more plausible candidate for AMD pathogenicity, we emphasize the pressing need for future studies to carefully isolate the potential roles of ARMS2 and HTRA1 in AMD pathogenesis to pinpoint the causal agent. In this review, we will focus on the evidence for HTRA1’s role in AMD pathogenesis and its interplay with aging, oxidative stress, and other genetic risk factors that predispose development of wet AMD [Figure 4].

### Evidence for HTRA1’s role in wet AMD

HTRA1 is a 51-kDa secreted protein that belongs to the high temperature requirement A (HtrA) family of chymotrypsin-like serine proteases, which includes the paralogs HTRA2, HTRA3, and HTRA4^[139,140]^. Originally discovered in *E. coli* as an integral component of the heat shock response^[141,142]^, HTRA1 is widely expressed in human tissue and has been implicated in many physiological processes, notably ECM remodeling^[120–122]^ and TGF-β signaling^[123–127]^. Since the AMD risk haplotype does not extend into the HTRA1 coding region, researchers have long suspected that chronic excess HTRA1 expression mediates ARMS2-HTRA1 genetic risk by altering ECM physiology at the blood-retina barrier. These changes may result from either proteolytic degradation of Bruch’s Membrane or dysregulation of signaling pathways - especially TGF-β - that compromise blood-retina barrier integrity and/or promote CNV. It is important to note that these processes are not mutually exclusive and may have varying relevance to disease progression in specific AMD patients.

The preceding mechanisms share the common assumption that the ARMS2-HTRA1 risk haplotype increases retinal expression of HTRA1 in AMD patients, but this assertion remains unsettled due to conflicting results. Early studies of HTRA1 expression noted excess HTRA1 levels in patients harboring the AMD risk haplotype, but the sample sizes were generally small (< 10 subjects)^[119,143–145]^. In contrast, later studies with larger patient cohorts (> 30 subjects) oftentimes did not observe a significant difference in HTRA1 expression between patients harboring the protective and risk haplotypes^[146–148]^. However, these apparently contradictory findings may be resolved by impinging environmental factors, chiefly oxidative stress. Recent studies have found that oxidative stress induces HTRA1 expression^[149–151]^. Oka and colleagues were the first to show that oxidative stress elicits HTRA1 expression in the human RPE cell line ARPE-19 and murine embryonic fibroblasts (MEF)^[150]^. Excess HTRA1 induced cellular senescence by activating the p38 mitogen-activated protein kinase (MAPK) pathway, which was dependent on HTRA1 proteolytic activity. In our previous study, ARPE-19 cells challenged at their apical surface with oxidized low-density lipoprotein, which mimics the shedding of highly oxidized photoreceptor outer segments, had significantly increased HTRA1 levels but surprisingly had no change in VEGF levels 24 h after stimulation^[151]^. In contrast, when these cells were exposed to secretions from macrophages under oxidative stress, both HTRA1 and VEGF levels increased, likely *via* the Wnt signaling cascade. These results highlight the role of oxidative stress in exacerbating HTRA1 expression associated with the AMD risk haplotype and the role of pro-angiogenic factors secreted by macrophages in accelerating neovascularization in the wet AMD eye.

Assuming HTRA1 expression is increased by the ARMS2-HTRA1 risk haplotype and/or under heightened oxidative stress, chronic excess HTRA1 would be predicted to compromise the structural integrity of the blood-retina barrier. HTRA1 is known to cleave many transmembrane and secreted proteins in the ECM, including critical components of Bruch’s Membrane. Major structural ECM proteins known to be substrates of HTRA1 proteolysis include decorin^[152,153]^, EGF-containing fibulin-like extracellular matrix protein 1 (EFEMP1)^[154]^, fibronectin^[122,153,155]^, fibromodulin^[120,152]^, nidogen-1^[122]^, nidogen-2^[122]^, thrombospondin-1^[143,151]^, type II collagen^[152]^, and vitronectin^[120]^. Gradual degradation of Bruch’s Membrane opens pathways for vascular penetrance from the choroid into the neural retina, heightening the risk of wet AMD development. An underappreciated aspect of this process is that the ECM fragment by-products of HTRA1 proteolytic digestion may themselves be pro-angiogenic, exacerbating risk for wet AMD beyond the breakdown of physical separation between the choroid and neural retina. For example, it was recently shown that the N-terminal fragment of thrombospondin-1 produced by HTRA1 digestion promotes endothelial tube formation, whereas the parent thrombospondin-1 is anti-angiogenic^[143]^. Presumably, the parallel processes of structural proteolysis and ECM fragment signaling always have the potential to mediate rapid breakdown of the blood-retina barrier. Future studies are needed to elucidate the regulatory mechanisms that keep these potentially deleterious processes within healthy limits and to identify the age-related or AMD risk factor-specific changes that disrupt these safeguards, leading to pathology.

HTRA1 also regulates TGF-β signaling, which serves diverse functions in adults and developing embryos^[156]^. The pathway is triggered by ligand binding to TGF-β receptors, followed by phosphorylation of SMAD proteins, which then form complexes that translocate into the nucleus and act as transcription factors^[156]^. HTRA1 has been implicated in cleaving multiple members of the TGF-β protein family, including mature TGF-β^[125]^ (although this is disputed^[123,126]^), proTGF-β1^[126]^, Type II and Type III TGF-β receptors^[123]^, and latent TGF-β binding protein 1 (LTBP1)^[127]^. Regardless of its mechanism of action, HTRA1 proteolytic activity decreases TGF-β signaling, leading researchers to explore the consequences of downregulated TGF-β on AMD pathogenesis. Zhang and colleagues suggested that excess HTRA1 promotes angiogenesis *via* growth differentiation factor 6 (GDF6), a member of the TGF-β family^[145]^. In patients harboring the ARMS2-HTRA1 risk variant rs10490924, they noted increased HTRA1 and decreased GDF6. Conversely, in HTRA1-knockout mice, they observed increased GDF6 and decreased VEGF levels, suggesting that the HTRA1-null allele is protective against VEGF-mediated retinal neovascularization. Additionally, a study of Type II TGF-β receptor knockout mice (TGFBR2^−/−^) at 3-weeks and 6-months exhibited clear signs of wet AMD, including deformed RPE, mononuclear cells in the subretinal space, and degraded photoreceptor outer segments^[157]^. Critically, VEGF was significantly increased in these mice, suggesting that insufficient TGF-β signaling can induce CNV. Schlecht and colleagues proposed that VEGF and TGF-β serve antagonistic roles in maintaining endothelial homeostasis^[157]^. Since HTRA1-mediated cleavage of either TGF-β or its receptors decreases overall TGF-β signaling, it is plausible that chronic excess HTRA1 induces CNV because there is insufficient TGF-β to counteract VEGF activity. However, more research is needed to identify the precise mechanism by which decreased TGF-β activity accelerates AMD pathogenesis.

### GENETIC INSIGHTS SUGGEST A NEW MODEL FOR WET AMD PROGRESSION

**Figure 5.**
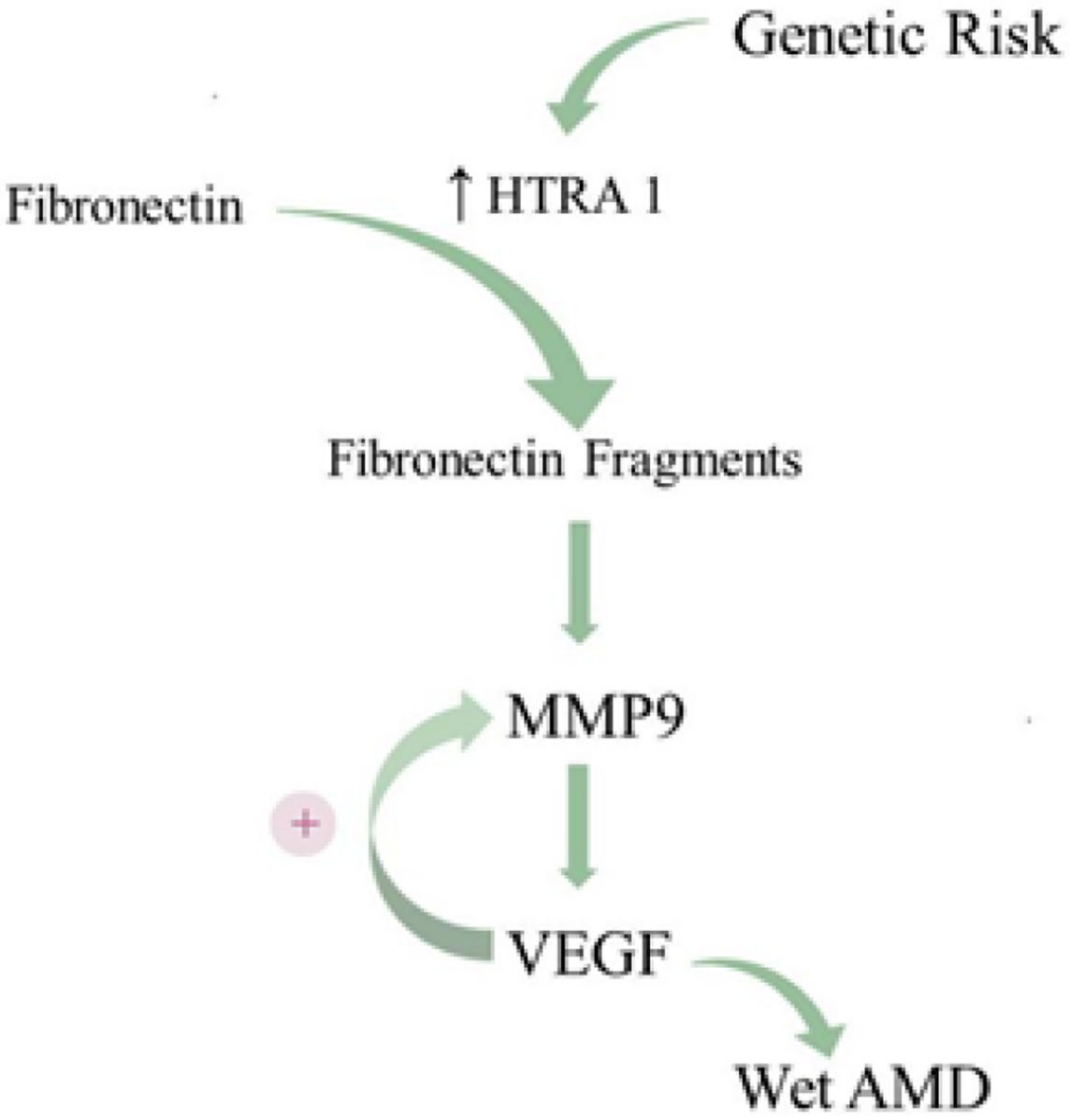
The proposed HTRA1-MMP9 axis in wet AMD. The ARMS2-HTRA1 risk haplotype increases expression of HTRA1, heightening the degradation of ECM constituents. HTRA1-mediated cleavage of the ECM protein fibronectin generates fibronectin fragments, which stimulate MMP9 expression. Positive feedback between MMP9 and VEGF promotes CNV and leads to wet AMD. AMD: age-related macular degeneration; ECM: extracellular membrane; VEGF: vascular endothelial growth factor; CNV: choroidal neovascularization

For years, research on AMD pathogenesis has largely focused on the two most prominent genetic risk loci as unveiled through GWAS: ARMS2-HTRA1 and CFH. However, critical perspectives can be gleaned by assessing genetic risk factors that predispose development to wet AMD *vs.* dry AMD. In the largest GWAS on AMD to date, Fritsche and colleagues found 52 variants distributed across 34 loci that are associated with progression to late-stage AMD, either dry or wet^[158]^. Interestingly, only four loci harbored variants that significantly predispose development to the wet subtype: ARMS2-HTRA1 on chromosome 10, CETP on chromosome 16, MMP9 on chromosome 20, and SYN3-TIMP3 on chromosome 22 (mutations in this locus are also associated with Sorby’s Fundus Dystrophy, an AMD-like phenotype). While these four loci predispose development to wet AMD over dry AMD, the MMP9 locus is unique in that its risk variant is *only* associated with wet AMD, *not* dry AMD. This region encodes matrix metalloproteinase 9 (MMP9), which, like HTRA1, is a critical regulator of ECM remodeling. MMP9 is known to participate in positive feedback with VEGF; when MMP9 levels increase, so do VEGF levels, and *vice versa*^[159]^. Even more striking, MMP9 expression in cultured RPE cells is elicited by exposure to fibronectin fragments, which notably are the byproduct of HTRA1-mediated degradation of fibronectin^[160]^. Chronically elevated fibronectin fragment levels along the blood-retina barrier due to excess HTRA1 expression would also be expected to increase MMP9 expression. Much like the pro-angiogenic N-terminal fragment of thrombospondin-1, excess MMP9 would not only accelerate Bruch’s Membrane breakdown, but also stimulate VEGF expression, thereby triggering neovascularization. Over time, elevated expression of HTRA1 in dry AMD patients harboring the ARMS2-HTRA1 risk haplotype may elicit sufficient MMP9 and, subsequently, VEGF to trigger the transition to wet AMD. While it remains unclear why only some dry AMD patients develop wet AMD, these findings suggest that the HTRA1-MMP9 axis may be the critical intermediate between ARMS2-HTRA1 genetic risk and wet AMD [Figure 5].

### CONCLUSION: NEW AVENUES FOR INVESTIGATION AND THERAPEUTIC INTERVENTION

Despite major advancements in our understanding of the risk factors for AMD through GWAS, eQTL, and longitudinal case studies, there is a dearth of effective treatment options for patients living with AMD. Currently, the only robustly effective treatment for wet AMD is anti-VEGF therapy (e.g., ranibizumab, bevacizumab). While anti-VEGF therapy has undoubtedly revolutionized clinical management of wet AMD, this approach targets a late downstream effector of CNV, requires repeated monthly injections to sustain therapeutic effect, and is ineffective in a substantial portion of wet AMD patients^[161,162]^. Above all, anti-VEGF offers no benefit to people living with dry AMD, who constitute roughly 90% of all AMD cases. The lack of novel and effective alternative approaches to treat both wet and dry AMD is a pressing unmet medical need facing ophthalmologists worldwide. Our analysis suggests that HTRA1 and MMP9 are strong candidate therapeutic targets that warrant further investigation. Because HTRA1 is considerably upstream of VEGF and is associated with both wet and dry AMD, anti-HTRA1 therapy holds promise of slowing or halting the progression of both wet and dry AMD. Our lab has uncovered encouraging preliminary evidence that anti-HTRA1 single-chain variable fragment (scFv) can significantly reduce the size of neovascular lesions in a laser-induced mouse model of wet AMD^[151]^. Further support for this approach comes from a recent report by Lill and colleagues, who independently generated an anti-HTRA1 Fab and demonstrated that Dickkopf-related protein 3 (DKK3) levels in the aqueous humor of patients with dry AMD could serve as a biomarker for HTRA1 proteolytic inhibition by their anti-HTRA1 Fab^[163]^. Despite these early successes, HTRA1’s position upstream of VEGF implies that inhibiting its proteolytic activity will affect a greater number of physiological pathways in the retina, increasing the likelihood of deleterious side effects. For this reason, MMP9 may be a viable alternative or combinatorial therapeutic target in patients with wet AMD who do not respond adequately to anti-VEGF alone. Regardless of which therapeutic approach is ultimately most successful, these investigations will yield valuable insights into the pathways leading from genetic risk to AMD pathology and potentially uncover new ways of conceptualizing and treating other neovascular diseases.

## Figures and Tables

**Table 1.** Comparison of risk factors and clinical features between AMD and related retinal dystrophies

Disease	Risk factors	Clinical features	Ref.
Age-related macular degeneration (AMD)	Age > 55 years is the greatest risk factor Genetic: strongly associated with variants in the *CFH* and *ARMS2-HTRA1* loci; other variants also implicated Environmental: cigarette smoking, high body mass index, other oxidative stressors	Development of drusen followed by geographic atrophy (GA) and/or choroidal neovascularization (CNV)	[5,6]
Polypoidal Choroidal Vasculopathy (PCV)	Like AMD, age > 55 years is a risk factor Genetic: associated with AMD risk variants in the *ARMS2-HTRA1, CFH, Elastin* and *C2* loci. Notably, only rs10490924, an ARMS2/LOC38771 coding variant, displays a statistically significant difference between PCV and AMD risk Environmental: cigarette smoking, high body mass index, other oxidative stressors	Abnormal branching network of blood vessels displaying polyps or polypoidal dilations within the Bruch’s Membrane in addition to choroidal thickening	[7–10]
Doyne Honeycomb Retinal Dystrophy	Genetic: R345W variant in *EFEMP1*, which encodes Fibulin-3, an ECM protein	Radial/honeycomb pattern of drusen formation leading into geographic atrophy and choroidal neovascularization	[11–13]
Sorby’s Fundus Dystrophy	Genetic: loss of function missense and nonsense variants in *TIMP3*, an MMP regulator	Accumulation of drusen in the subretinal space preceding subretinal hemorrhaging; RPE detachment and atrophy; and possibly choroidal neovascularization	[14,15]
Stargardt Disease	Genetic: 95% of cases are attributed to variants in *ABCA4*, an ATP-binding cassette transporter protein. The remaining 5% of cases are associated with variants in *STGF4, ELOVL4*, and *PRPH2*	Accumulation of A2E and lipofuscin in RPE cells, leading to dyschromatopsia and macular degeneration. Several variants are associated with “fovea-sparing”	[16,17]
Best Vitelliform Macular Dystrophy	Genetic: variants in *BEST1*, encoding an ion channel protein that regulates Ca^2+^ signaling	Lipofuscin accumulation within the RPE and macular vitelliform lesions, leading to cellular atrophy and sometimes choroidal neovascularization	[18,19]
Angioid (Knapp) Streaks	Phenotype associated with Pseudoxanthoma Elasticum, Paget Disease, Ehlers-Danlos Syndrome, sickle cell hemoglobinopathies, and other diseases	Calcification of Bruch’s Membrane, leading to the development of linear breaks, lesions, and choroidal neovascularization	[20–22]

**Table 2.** Inflammatory agents implicated in wet AMD progression

Protein	Location	Function	Ref.
IL-6	Soluble	Induces expression of angiogenic cytokines, including VEGF; regulates CNV progression	[62–64]
IL-8	Soluble	Pro-angiogenic factor; strong chemotactic factor for neutrophils; elicits VEGF-A and VEGFR2 expression by endothelial cells	[65–67]
MCP-1/ CCL2	Soluble	Regulates migration and infiltration of monocytes and macrophages; participates in VEGF_-_induced angiogenesis; and contributes to the formation of sub-foveal choroidal neovascular membranes	[63,68–70]
CCR2	Membrane	Promotes CNV formation by enhancing recruitment of myeloid cells	[68]
ICAM-1	Membrane	Associates with inflammatory cells in subretinal neovascular lesions	[71]
VEGF	Soluble	Most prominent pro-angiogenic factor that stimulates CNV	[72]
IP-10	Soluble	Associated with CNV	[73]
TGF-β	Soluble	Both promotes and inhibits CNV via diverse signaling pathways	[74,75]
IFN-γ	Soluble	Anti-angiogenic cytokine; induces expression of CFH and major histocompatibility complex-II in RPE cells	[76,77]
IGF-1	Soluble	Pro-angiogenic cytokine; promotes CNV	[78,79]

IL: interleukin; CCL2:C-C motif chemokine ligand 2; MCP1: monocyte chemotactic protein 1; ICAM1: intercellular adhesion molecule 1; VEGF: vascular endothelial growth factor; IP-10: interferon gamma-inducible protein-10; TGF-β: transforming growth factor β; IFN-γ: interferon γ; IGF-1: insulin-like growth factor-1; AMD: age-related macular degeneration; CNV: choroidal neovascularization
